# Cyanidin-3-*O*-glucoside inhibits the β-catenin/MGMT pathway by upregulating miR-214-5p to reverse chemotherapy resistance in glioma cells

**DOI:** 10.1038/s41598-022-11757-w

**Published:** 2022-05-11

**Authors:** Yuan Zhou, Li Chen, Deping Ding, Ziheng Li, Li Cheng, Qiuyun You, Shunbo Zhang

**Affiliations:** 1 College of Pharmacy, Hubei University of Chinese Medicine, No. 16 Huangjiahu West Road, Huangjiahu University Town, Hongshan District, Wuhan, 430065 Hubei China; 2 Department of Pharmacy, Taihe Hospital, Hubei University of Medicine, Shiyan, 442000 Hubei China; 3 Department of Infectious Diseases, Taihe Hospital, Hubei University of Medicine, Shiyan, 442000 Hubei China

**Keywords:** Cancer, Cell biology, Molecular biology

## Abstract

Overcoming resistance to alkylating agents has important clinical significance in glioma. Cyanidin-3-*O*-glucoside (C3G) has a tumor-suppressive effect on tumor cells. However, whether it plays a role in temozolomide resistance in glioma is still unclear. We constructed a TMZ-resistant LN-18/TR glioma cell line, observed the effect of C3G on TMZ resistance in this cell line, and explored the role of miR-214-5p in chemoresistance. Results showed that β-catenin and MGMT were significantly upregulated in LN-18/TR cells. C3G upregulated miR-214-5p and enhanced the cytotoxic effect of temozolomide on LN-18/TR cells. Contrarily, C3G downregulated β-catenin and MGMT. Moreover, the miR-214-5p mimic downregulated β-catenin and MGMT in LN-18/TR cells, whereas the miR-214-5p inhibitor had the opposite effect; the miR-214-5p inhibitor significantly blocked the C3G-induced downregulation of β-catenin and MGMT. C3G or the miR-214-5p mimic enhanced temozolomide-induced apoptosis in LN-18/TR cells, whereas the miR-214-5p inhibitor blocked this effect. Furthermore, C3G or miR-214-5p agomir combined with TMZ significantly inhibited the growth of LN-18/TR tumors. Collectively, our research discovered the potential signaling mechanism associated with C3G-mediated suppression of TMZ resistance in LN-18/TR cells through miR-214-5p, which can facilitate the treatment of MGMT-induced resistance in glioma cells.

## Introduction

Glioma originates from the inner constituent cells of the brain and is a primary tumor of the central nervous system^1^. The current treatment for glioma is comprehensive treatment based on surgery, radiotherapy, and chemotherapy^2^. Because glioma is located in the brain, surgery is difficult, and it is difficult for drugs to pass through the blood–brain barrier, which complicates treatment. This is especially true for high-grade glioma, which progresses quickly and has a poor prognosis, presenting a major challenge for clinical treatment^3,4^. Alkylating agents have mild side effects and overcome the limitations of traditional chemotherapeutic drugs that do not easily penetrate the blood–brain barrier. Therefore, they have become important drugs for the treatment of glioma, with temozolomide (TMZ) being the most representative^5,6^. However, as with other chemotherapeutics, patients often develop acquired resistance after treatment with alkylating agents^5,6^. Acquired drug resistance often leads to failed alkylating agent treatment, which is also one of the main causes of patient death. Therefore, overcoming the resistance to alkylating agents has important clinical significance for the treatment of glioma.

**Figure 1:**
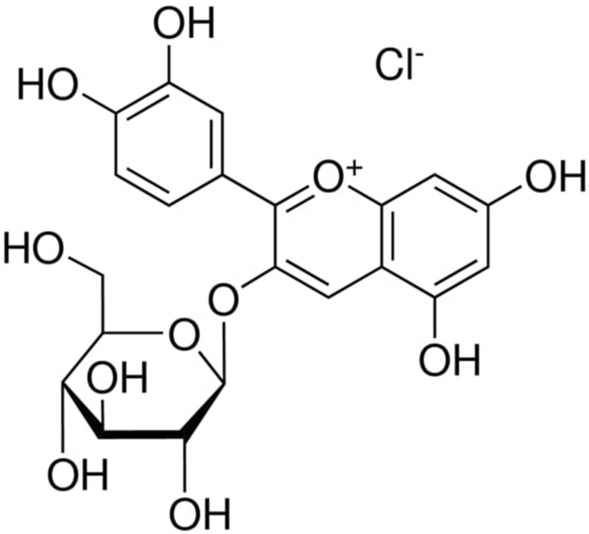
Molecular structure of cyanidin-3-*O*-glucoside (C3G).

Cyanidin-3-*O*-glucoside (C3G) is an anthocyanin and a water-soluble flavonoid compound widely found in plants, and mainly in blueberries, black rice, and purple potatoes^7,8^. The molecular formula of C3G is C_21_H_21_O_11_, and the molecular structure is shown in Fig. 1. Its basic structure contains two benzene rings and a large number of phenolic hydroxyl groups^9^. Because of the unique molecular structure of C3G, it has a strong antioxidant effect^9^. Previous studies have shown that C3G has anti-inflammatory, anti-tumor, and fat metabolism roles^10^. C3G has been confirmed to have a tumor-suppressive effect in breast cancer^11^, colon cancer^11,12^, osteosarcoma^13^, and other malignant tumors, highlighting the relatively high development value of C3G. However, the effect of C3G on chemotherapy resistance in glioma is still unclear, and further research is needed.

In recent years, microRNAs (miRNAs) have been found to play important roles in the occurrence and development of malignant tumors. miRNAs are generally 20–25 nt in length and play physiological roles through the post-transcriptional regulation of mRNAs^14^. Many miRNAs cause mRNA degradation through specific base pairing with target mRNAs, ultimately affecting the expression of the encoded proteins^15,16^. Some miRNAs are abnormally expressed and play a role in the resistance of tumor cells to chemotherapeutics^17–19^. miR-214-5p has a tumor-suppressive effect in a variety of malignant tumors mediated by inhibiting tumor cell proliferation, migration, invasion, and other malignant biological behaviors^20,21^. However, whether miR-214-5p plays a role in TMZ resistance in glioma is still unclear. This study aimed to construct a TMZ-resistant glioma cell line, observe the effect of C3G on TMZ resistance in these cells, and explore the role of miR-214-5p in chemoresistance. This will provide theoretical support for the clinical treatment of glioma.

## Results

### β-Catenin and *O*-6-methylguanine-DNA methyltransferase (MGMT) are upregulated in LN-18/TR cells

**Figure 2:**
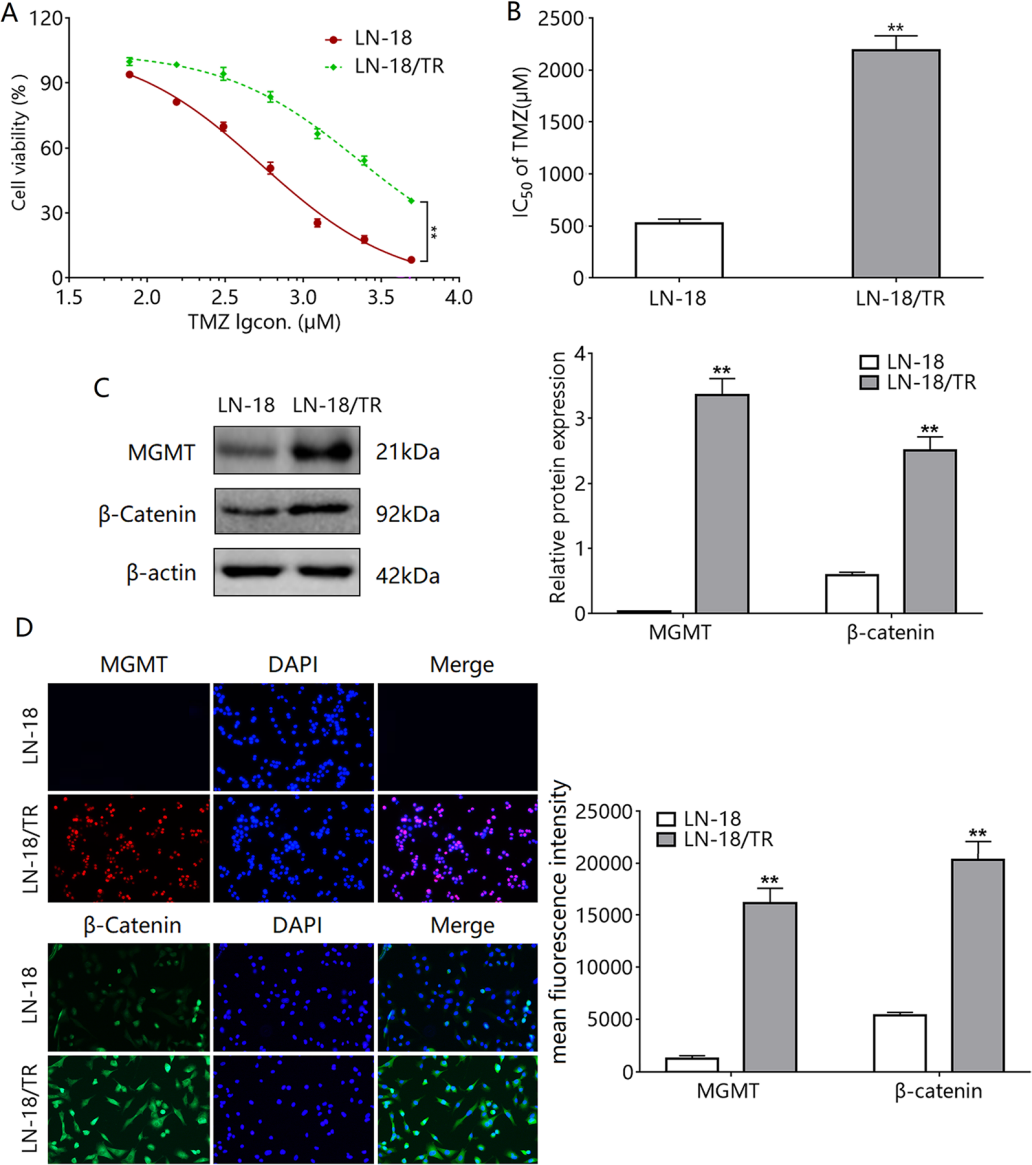
Expression of β-catenin and MGMT in LN-18/TR cells. (**A**) LN-18 and LN-18/TR cells were treated with temozolomide (TMZ; 80, 160, 320, 640, 1280, and 2560 μM) for 72 h, and the cell proliferation inhibition rate was detected by the MTT method. (**B**) IC_50_ of TMZ in cells. (**C**) Western blotting was performed to detect β-catenin and MGMT protein expression in cells. (**D**) Immunofluorescence was performed to detect β-catenin and MGMT protein expression in LN-18 and LN-18/TR cells. **P < 0.01 compared to LN-18 cells.

To study the role of C3G in TMZ resistance in glioma cells, we used LN-18 cells to construct the TMZ-resistant glioma cell line LN-18/TR. LN-18/TR cells had significantly enhanced resistance to TMZ (Fig. 2A, B). The resistance index of LN-18/TR cells to TMZ was 4.51. The drug resistance protein MGMT plays an important role in glioma cells; therefore, we examined the expression changes of MGMT and its upstream signal protein β-catenin. The expression of β-catenin and MGMT were significantly higher in LN-18/TR cells than in LN-18 cells (Fig. 2C, D).

### C3G induces TMZ sensitization and upregulation of miR-214-5p in LN-18/TR cells

**Figure 3:**
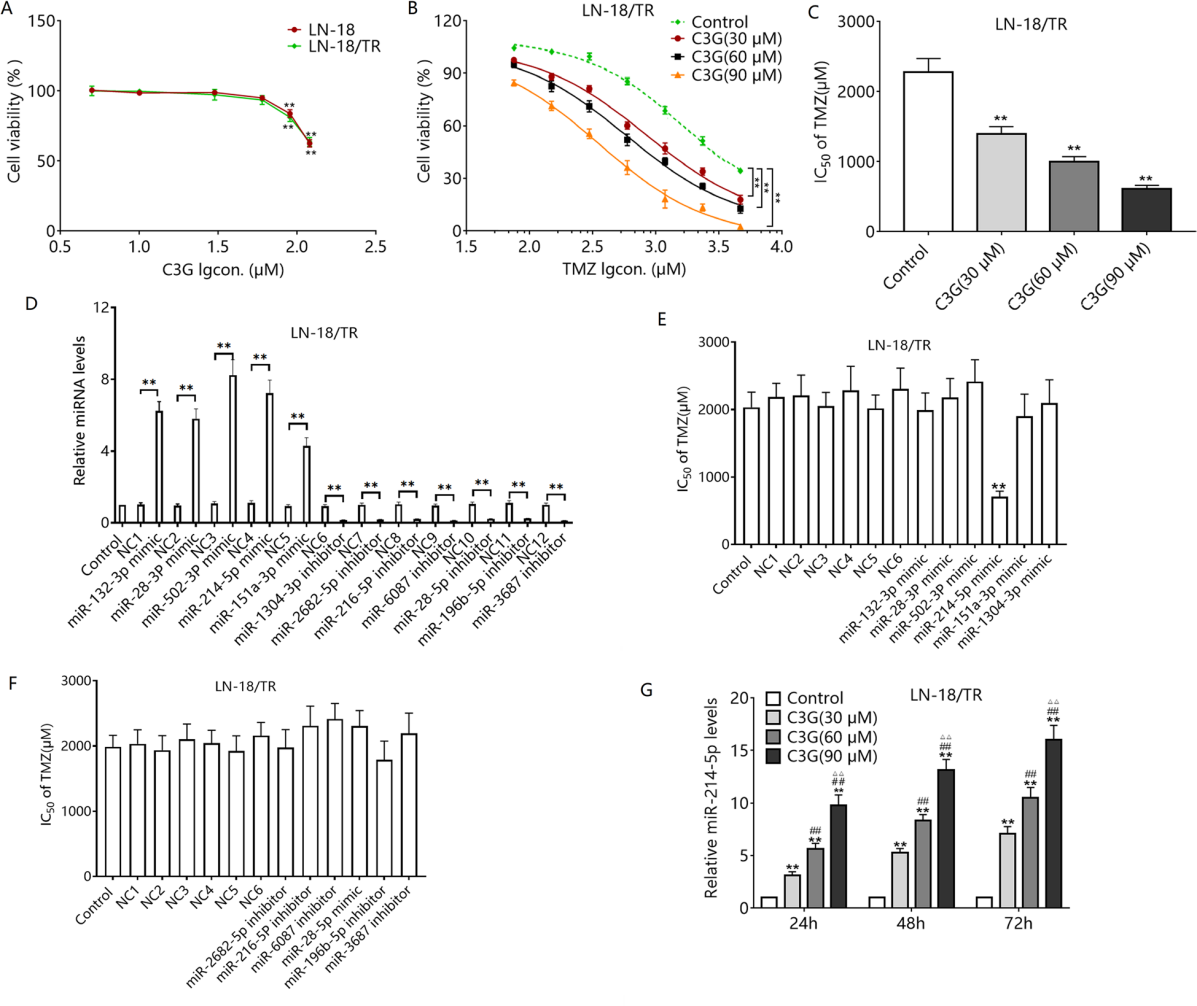
Cyanidin-3-*O*-glucoside (C3G) induces sensitization to temozolomide (TMZ) and the upregulation of miR-214-5p in LN-18/TR cells. (**A**) LN-18 and LN-18/TR cells were treated with C3G (5, 10, 30, 60, 90, and 120 μM) for 72 h, and the cell proliferation inhibition rate was detected by the MTT method. (**B**) LN-18 and LN-18/TR cells were treated with C3G (30, 60, and 90 μM) combined with TMZ (80, 160, 320, 640, 1280, and 2560 μM), and the cell proliferation inhibition rate was detected by the MTT method. (**C**) The IC_50_ of TMZ in LN-18/TR cells treated with 30, 60, and 90 μM C3G. (**D**) LN-18/TR cells were transfected with mimics of miR-132-3p, miR-28-3p, miR-502-3p, miR-214-5p, miR-151a-3p, and miR-1304-3p, or inhibitors of miR-2682-5p, miR-216-5p, miR-6087, miR-28-5p, miR-196b-5p, and miR-3687, and corresponding negative controls. The expression levels of each miRNA were detected using RT-FqPCR. **P < 0.01. (**E**) IC50s of TMZ in LN-18/TR cells transfected with each miRNA mimic. **P < 0.01 compared to NC4. (**F**) IC50s of TMZ in LN-18/TR cells transfected with each miRNA inhibitor. (**G**) LN-18/TR cells were treated with 30, 60, and 90 μM C3G, and the expression of miR-214-5p was assessed by RT-FqPCR. **P < 0.01 compared to control.

To use C3G rationally, we first observed the effect of C3G on the proliferation of LN-18 and LN-18/TR cells. The results showed that there was no significant difference between LN-18 and LN-18/TR cells with regard to the effect of C3G (5–150 μM) on proliferation. Lower concentrations of C3G (5–60 µM) had little effect on the proliferation of either LN-18 or LN-18/TR cells (Fig. 3A), whereas higher concentrations (90–120 μM) significantly reduced the proliferation ability of both LN-18 and LN-18/TR cells (Fig. 3A). To avoid the interference of C3G on cell proliferation, we used 30, 60, and 90 μM of C3G for further experiments. TMZ combined with 30, 60, and 90 μM C3G had a greater inhibitory effect on LN-18/TR cell viability than TMZ alone (Fig. 3B, C). Our preliminary experiments found that C3G could induce the up-regulation of miR-132-3p, miR-28-3P, miR-502-3P, miR-214-5p, miR-151a-3p, and miR-1304-3p expression in LN-18/TR cells. Meanwhile, C3G could induce down-regulation of miR-2682-5p, miR-216-5p, miR-6087, miR-28-5p, miR-196b-5p, and miR-3687 expression (Fig. S1). LN-18/TR cells were transfected with miRNA mimics or inhibitors of these miRNAs, respectively. Among them, only the miR-214-5p mimic elicited strong TMZ sensitization (Fig. 3D–F). RT- FqPCR validation found that treatment with 30, 60, and 90 μM C3G for 24, 48, and 72 h concentration-dependently upregulated miR-214-5p in LN-18/TR cells (Fig. 3G).

### *CTNNB1* has miR-214-5p-binding sequences

**Figure 4:**
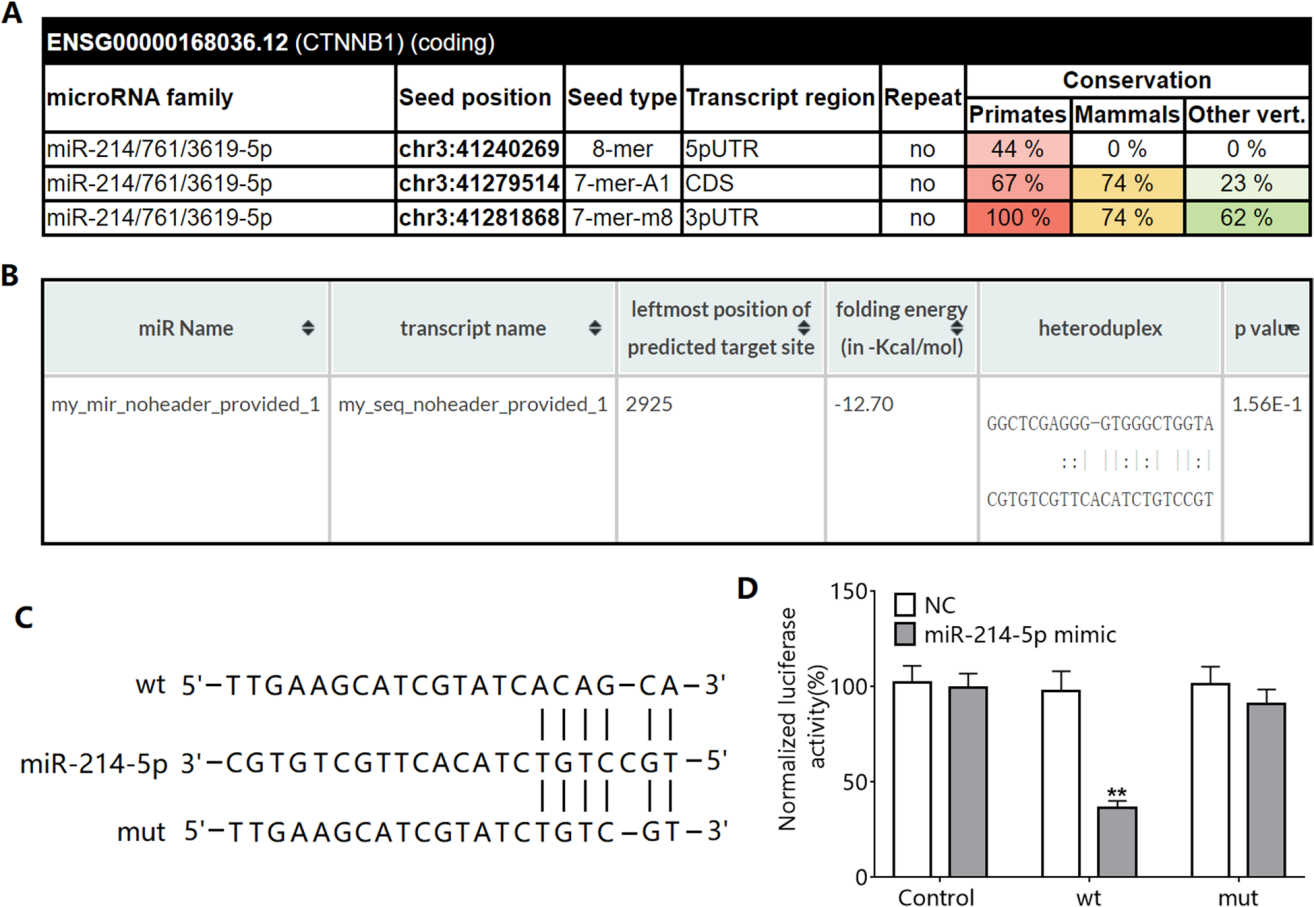
Bioinformatics prediction of the binding of *CTNNB1* to miR-214-5p. (**A**) miRcode predicted miR-214-5p-binding sites in *CTNNB1* mRNA and the conservation of the binding sites among species. (**B**) RNA22 v2 predicted the sequence of the miR-214-5p-binding site in *CTNNB1* mRNA. (**C**) 3′-UTR of *CTNNB1* mRNA and miR-214-5p seed region complementary paired sequence. (**D**) The dual luciferase reporter gene experiment verifies the interaction between miR-214-5p and 3′-UTR of *CTNNB1* mRNA and the functions. **P < 0.01 compared to NC. *CTTNB1*: gene encoding the β-catenin protein.

To confirm whether miR-214-5p can target CTNNB1 (the gene encoding β-catenin), we conducted bioinformatics analysis. The results of the miRcode analysis showed that *CTNNB1* had a miR-214-5p-binding site, and the sequence of the site was relatively conserved among species (Fig. 4A). RNA22 v2 analysis also revealed that *CTNNB1* mRNA and miR-214-5p had potential interaction sites (Fig. 4B). Additionally, we found that the 3′-untranslated region (3′-UTR) of *CTNNB1* mRNA has a complementary pairing sequence with the seed region of miR-214-5p (Fig. 4C). A dual luciferase reporter gene experiment revealed that the luciferase activity of the cells transfected with plasmid containing miR-214-5p mimic and 3′-UTR of the wild-type *CTNNB1* mRNA was significantly reduced (Fig. 4D).

### C3G downregulates β-catenin and MGMT in LN-18/TR cells through miR-214-5p

**Figure 5:**
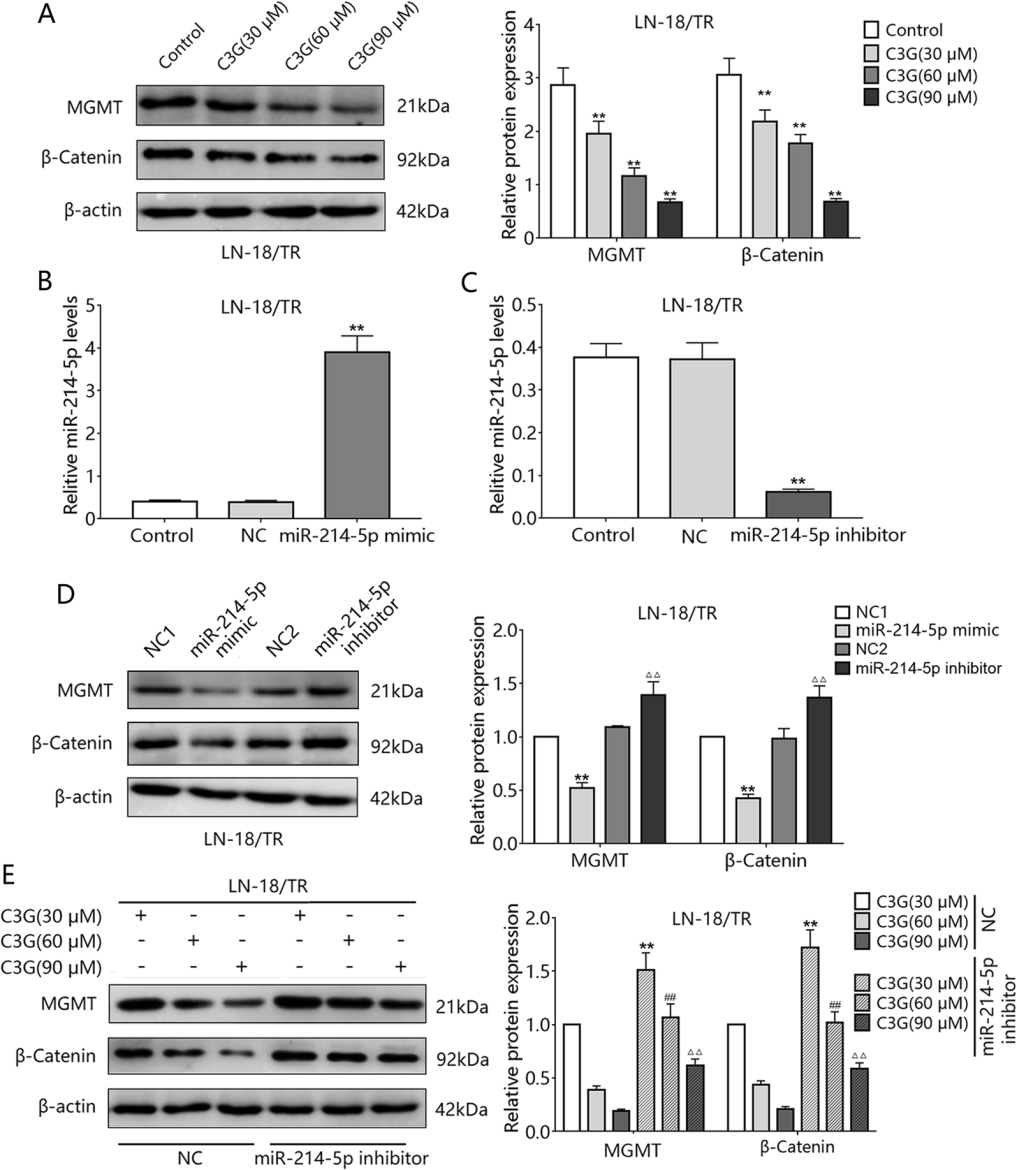
Cyanidin-3-*O*-glucoside (C3G) inhibits the β-catenin/MGMT signaling pathway through miR-214-5p. (**A**) LN-18/TR cells were treated with C3G (30, 60, and 90 μM) for 48 h, and β-catenin and MGMT protein expression was detected in the cell protein extracts. **P < 0.01 compared to the control. (**B**) RT-FqPCR was performed to detect miR-214-5p levels in miR-214-5p mimic or negative control (NC) groups. **P < 0.01 compared to NC. (**C**) RT-FqPCR was conducted to detect miR-214-5p levels in miR-214-5p inhibitor or NC groups. **P < 0.01 compared to NC. (**D**) Following promotion or inhibition of the miR-214-5p expression/activity in LN-18/TR cells, β-catenin and MGMT expression was detected in the cell protein extracts. **P < 0.01 and ^△△^*P* < 0.01 compared to NC1 and NC2, respectively. (**E**) LN-18/TR cells were treated with 30, 60, or 90 μM C3G for 48 h after transfection of NC or miR-214-5p mimic, and β-catenin and MGMT expression was detected in the cell protein extracts. **P < 0.01, ^##^*P* < 0.01, ^△△^*P* < 0.01 compared to 30, 60, and 90 μM C3G, respectively.

To verify whether C3G downregulates β-catenin protein expression through miR-214-5p, we conducted related molecular experiments. The results showed that treatment with 30, 60, and 90 μM C3G decreased β-catenin and MGMT expression in LN-18/TR cells (Fig. 5A). Since C3G-induced miR-214-5p might target *CTNNB1*, we assessed the effect of miR-214-5p on the expression of β-catenin and MGMT. The miR-214-5p mimic increased miR-214-5p levels in LN-18/TR cells, whereas the miR-214-5p inhibitor decreased the levels (Fig. 5B, C). Furthermore, the miR-214-5p mimic decreased β-catenin and MGMT levels in LN-18/TR cells, whereas the miR-214-5p inhibitor increased their levels (Fig. 5D). To further verify the role of miR-214-5p in C3G-mediated β-catenin and MGMT downregulation, we assessed the effect of C3G on the expression of β-catenin and MGMT proteins in LN-18/TR cells transfected with a miR-214-5p inhibitor. The miR-214-5p inhibitor blocked the ability of 30, 60, and 90 μM C3G to decrease β-catenin and MGMT levels in LN-18/TR cells (Fig. 5E).

### C3G inhibits resistance to TMZ through miR-214-5p

**Figure 6:**
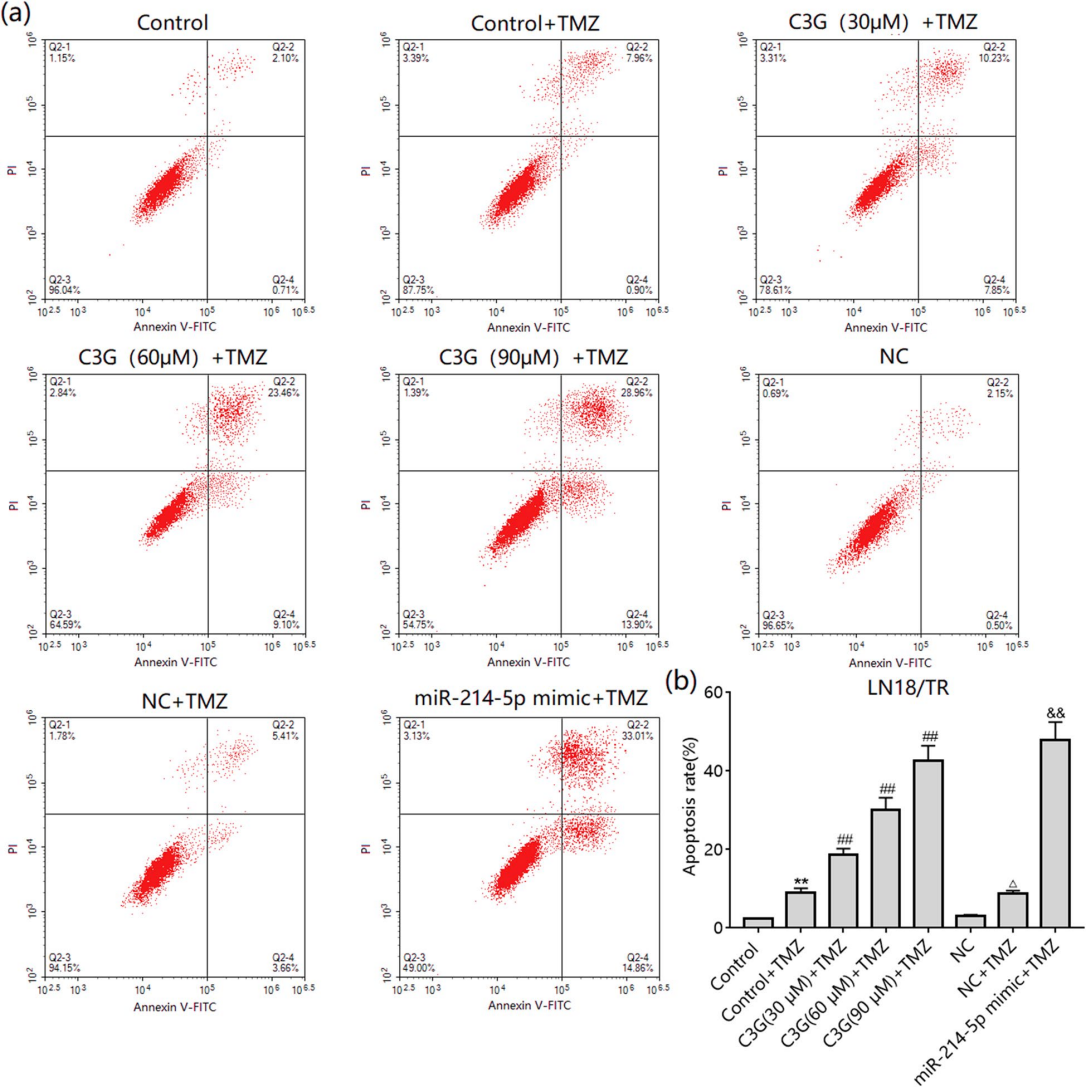
Cyanidin-3-*O*-glucoside (C3G) and miR-214-5p enhances temozolomide (TMZ)-induced apoptosis in LN-18/TR cells. (**a**) LN-18/TR cells were treated with 1000 µM TMZ alone or TMZ plus 30, 60, or 90 μM C3G, NC, or miR-214-5p mimic for 72 h. Flow cytometry was performed to detect apoptosis after each treatment. (**b**) The total apoptosis rate after each treatment. **P < 0.01 compared to the control; ^##^*P* < 0.01 compared to control + TMZ; ^△^*P* < 0.05; ^&&^*P* < 0.01 compared to NC + TMZ.

**Figure 7:**
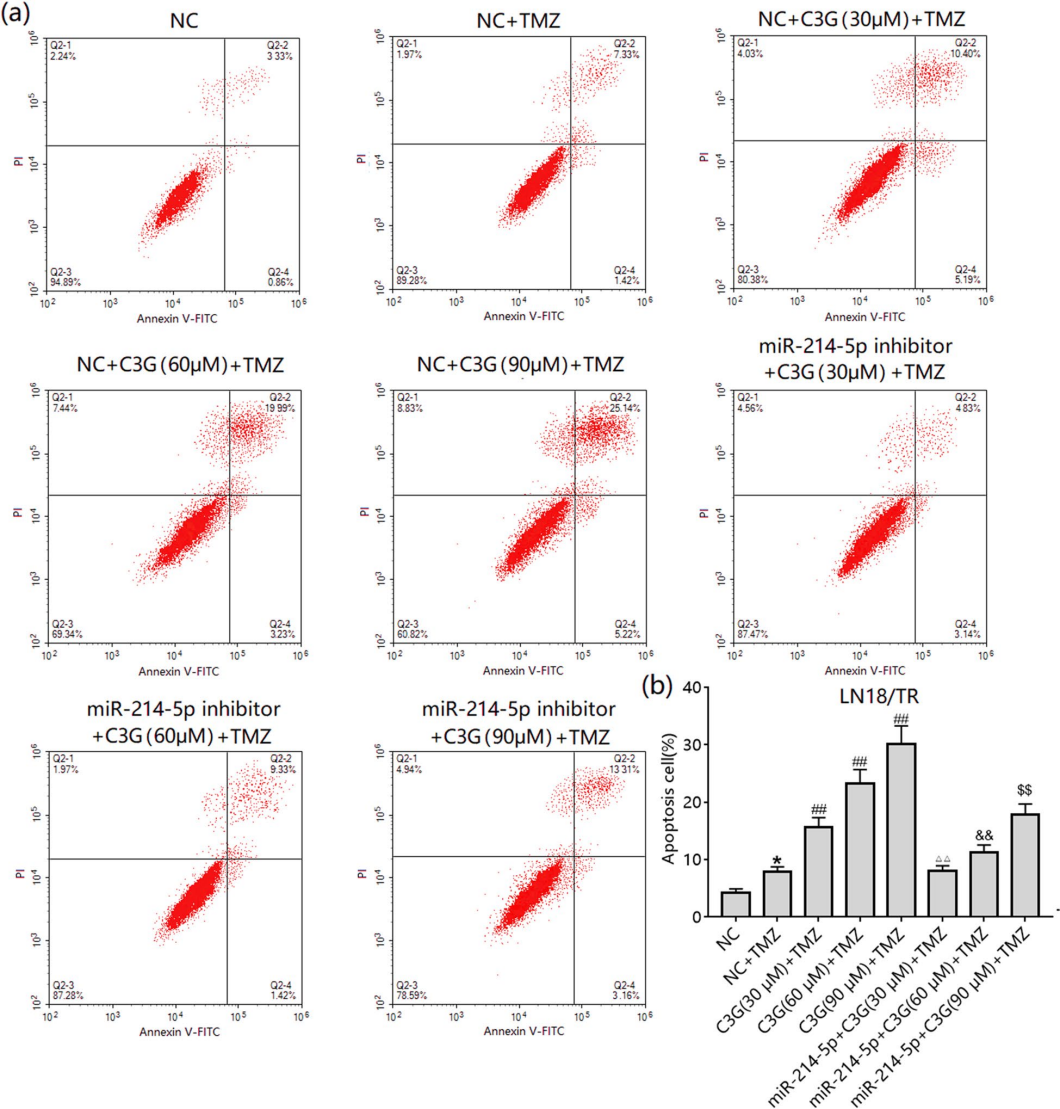
Cyanidin-3-*O*-glucoside (C3G) enhances the sensitivity of LN-18/TR cells to temozolomide (TMZ) by upregulating miR-214-5p. (**a**) LN-18/TR cells transfected with NC or miR-214-5p inhibitor were treated with 1000 μM TMZ or TMZ plus 30, 60, or 90 μM C3G for 72 h. Flow cytometry was performed to detect apoptosis after each treatment. (**b**) The total apoptosis rate after each treatment. *P < 0.05 compared to the control; ^##^*P* < 0.01 compared to NC + TMZ; ^△△^*P* < 0.01 compared to 30 μM C3G + TMZ; ^&&^*P* < 0.01 compared to 60 μM C3G + TMZ; ^$$^*P* < 0.01 compared to 90 μM C3G + TMZ.

We further observed the effect of C3G/miR-214-5p on TMZ-induced apoptosis. TMZ could weakly induce apoptosis in LN-18/TR cells, although treatment with 30, 60, and 90 μM C3G enhanced TMZ-induced apoptosis (Fig. 6a, b). miR-214-5p mimic transfection also enhanced TMZ-induced apoptosis in LN-18/TR cells (Fig. 6a, b). To observe the role of miR-214-5p in C3G-enhanced TMZ-induced apoptosis in LN-18/TR cells, we transfected the miR-214-5p inhibitor into LN-18/TR cells and assessed apoptosis after treatment with C3G and TMZ. The results showed that the miR-214-5p inhibitor blocked the effect of C3G on TMZ-induced apoptosis in LN-18/TR cells (Fig. 7a, b).

### C3G and miR-214-5p promote TMZ sensitivity in vivo

**Figure 8:**
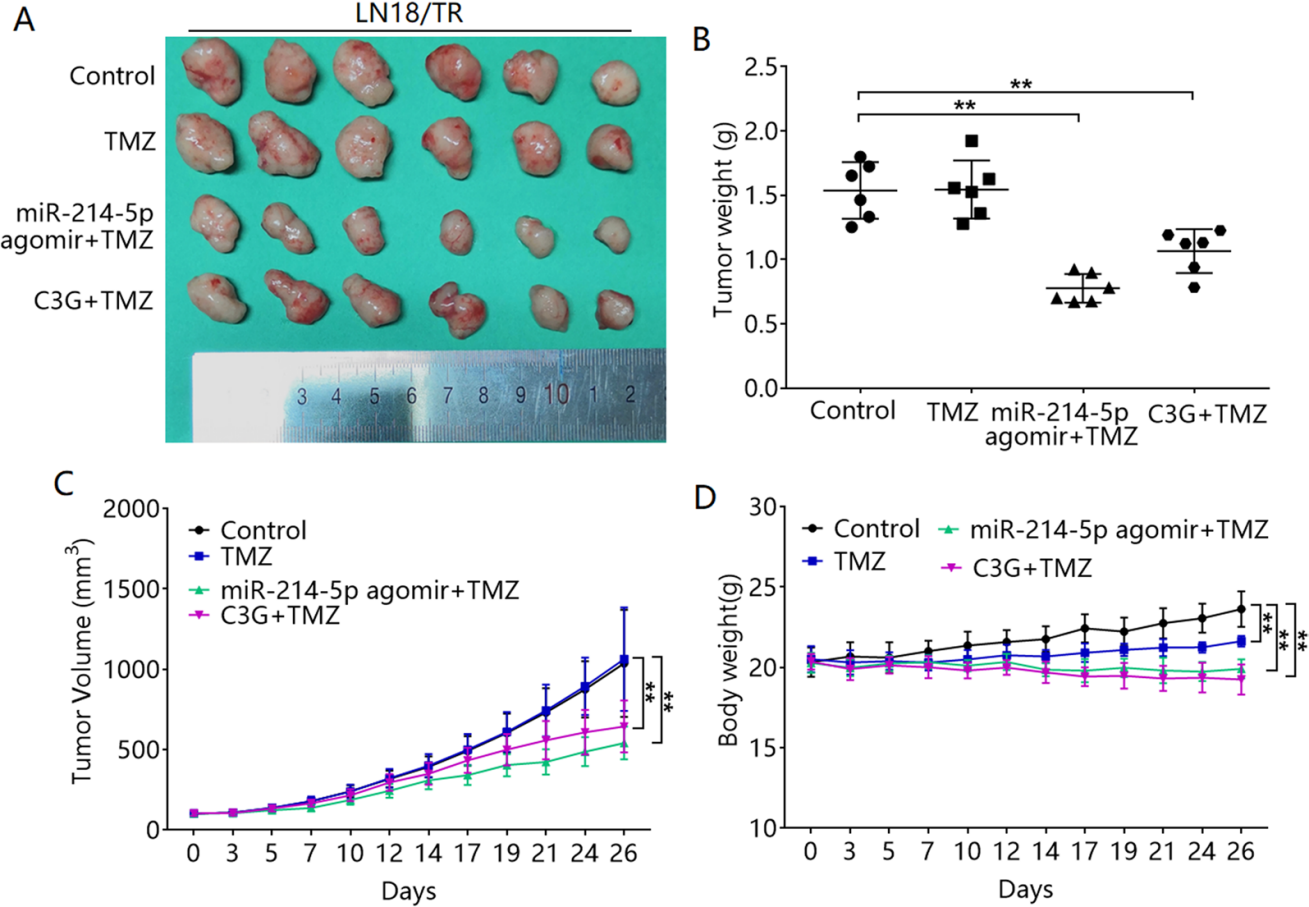
Cyanidin-3-*O*-glucoside (C3G) and miR-214-5p promote temozolomide (TMZ) sensitivity in vivo. (**A**) Representative images of control, TMZ, C3G + TMZ, miR-214-5p agomir + TMZ group tumors. (**B**) Tumor weights of each group of animals. **P < 0.01. (**C**) The tumor volume changes in each group of animals. Compared with control, **P < 0.01. (**D**) Changes in body weights of animals in each group. Compared with control, **P < 0.01. Compared with TMZ, ^##^*P* < 0.01.

We further observed the effects of C3G and miR-214-5p on TMZ-resistant LN-18/TR xenografts. In vivo results showed that TMZ had no obvious growth inhibitory effect on LN-18/TR tumors (Fig. 8A–C). C3G or miR-214-5p agomir combined with TMZ significantly inhibited the growth of LN-18/TR tumors (Fig. 8A–C). However, at the end of the experiment, the body weights of the mice in the TMZ group were significantly lower than those in the control group, whereas the body weights of the mice in the C3G combined with TMZ or miR-214-5p agomir combined with TMZ group were significantly lower than those in the TMZ group (Fig. 8D).

## Discussion

In this study, we successfully constructed a TMZ-resistant glioma cell line (LN-18/TR) through intermittent administration of TMZ and a gradual increase in its concentration. The resistance index of LN-18/TR cells to TMZ was 4.51. We found that levels of β-catenin and MGMT were significantly upregulated in LN-18/TR cells compared with those in the parental (non-resistant) cells. β-catenin is a functional protein encoded by *CTNNB1* that can form a protein complex with the adhesion protein cadherin to participate in cell adhesion^22,23^. β-catenin is also an important signaling molecule in the classic Wnt signaling pathway that participates in the regulation of cell proliferation, differentiation, and apoptosis^24,25^. β-catenin plays an important role in the resistance of various malignant tumors to chemotherapy drugs, immunosuppressants, and targeted drugs^26–28^. MGMT is a unique DNA repair protein found in bacteria and mammals that can repair DNA damage caused by alkylation^29^. Under normal circumstances, MGMT protects normal tissues from the damage of alkylating agents, whereas in tumor tissues, it increases resistance to alkylating chemotherapeutics^30,31^. Previous studies have shown that MGMT is a downstream target of Wnt/β-catenin and can mediate Wnt/β-catenin-induced TMZ resistance^32^. Bi et al.^33^ found that β-catenin can upregulate MGMT to maintain TMZ resistance in glioma cells, whereas cordycepin can inhibit the β-catenin/MGMT pathway to alleviate TMZ resistance. These findings suggest that the resistance of the LN-18/TR cells to TMZ is related to β-catenin/MGMT signaling.

C3G, a flavonoid widely found in plants, has been proven to exert a tumor-suppressive effect in malignant tumors. Our study found that C3G can sensitize LN-18/TR cells to TMZ. We also showed that C3G can upregulate miR-214-5p in LN-18/TR cells. Bioinformatics analysis showed that *CTNNB1* (the gene encoding β-catenin) and miR-214-5p have multiple potential interaction sites, indicating that *CTNNB1* could be a target of miR-214-5p. Therefore, we speculated that C3G might downregulate β-catenin through miR-214-5p, thereby sensitizing LN-18/TR cells to TMZ.

For further verification, we analyzed the molecular mechanism underlying the relationship among C3G, miR-214-5p, β-catenin, and MGMT. We found that C3G decreased β-catenin and MGMT expression in LN-18/TR cells. A miR-214-5p mimic and inhibitor exerted negative and positive regulatory effects, respectively, on the expression of β-catenin and MGMT in LN-18/TR cells. In addition, a miR-214-5p inhibitor blocked the C3G-induced downregulation of β-catenin and MGMT in LN-18/TR cells. These results suggest that C3G targets β-catenin through miR-214-5p, thereby inhibiting the expression of MGMT. We then assessed the effect of C3G and miR-214-5p on TMZ-induced apoptosis in LN-18/TR cells. Both C3G and the miR-214-5p mimic enhanced TMZ-induced apoptosis in LN-18/TR cells. In contrast, the miR-214-5p inhibitor blocked the effect of C3G on TMZ-induced apoptosis in LN-18/TR cells. Therefore, C3G enhances TMZ-induced apoptosis in LN-18/TR cells through miR-214-5p. In vivo results showed that C3G or miR-214-5p agomir enhanced the response of LN-18/TR xenograft tumors to TMZ. However, C3G or miR-214-5p agomir combined with TMZ caused severe weight loss in nude mice with xenograft tumors. It is suggested that the toxic and side effects of the two might be relatively strong, and corresponding chemical modification or preparation methods need to be improved. Based on these results, we hypothesized that C3G might upregulate miR-214-5p and inhibit β-catenin, thereby inhibiting the expression of MGMT, which leads to TMZ sensitization in LN-18/TR cells. Our research has discovered the potential signaling mechanism associated with C3G-mediated suppression of TMZ resistance in LN-18/TR cells through miR-214-5p, which can facilitate the treatment of MGMT-induced resistance in glioma cells.

In conclusion, our study confirmed that C3G has a TMZ-chemosensitization effect in glioma cells, which is related to the upregulation of miR-214-5p. miR-214-5p downregulates β-catenin, potentially via direct binding to *CTTNB1*, which downregulates MGMT and ultimately sensitizes cells to TMZ. Therefore, C3G might be a potential novel therapeutic drug for TMZ-resistant gliomas.

## Materials and methods

### Construction of the TMZ-resistant glioma cell line LN-18/TR and cell culture

LN-18 cells were purchased from Culture Collection of the Chinese Academy of Science (China). LN-18 cells were cultured in DMEM (high glucose) medium (Invitrogen, Carlsbad, CA, USA) containing 15% fetal bovine serum (Gibco-BRL, Grand Island, NY, USA), 50 U penicillin, and 50 μg streptomycin (HyClone, Logan, UT, USA). To create the LN-18 resistant cell line (LN-18/TR), LN-18 cells were cultured in DMEM complete medium with increasing concentrations of TMZ (Sigma-Aldrich, St. Louis, MO, USA; 80–2560 μM). The drug resistance index (IC_50_ of TMZ in LN-18/TR cells/IC_50_ of TMZ in LN-18 cells) of LN-18/TR cells was 4.51. All cells were cultured in a cell incubator at 37 °C, with 5% carbon dioxide, and 95% air.

### MTT analysis

Cells were diluted to a concentration of 6 × 10^4^ cells/mL and seeded in a 96-well plate at 100 μL/well. After drug (TMZ, C3G, or TMZ + C3G) treatment, the cells were treated with MTT (Solarbio, China) according to the manufacturer’s protocol. Briefly, 100 μL DMSO was added to each well and after shaking for 5 s, the absorbance was measured at 450 nm.

### Western blotting

After drug (C3G, miR-214-5p mimic, or miR-214-5p inhibitor) treatment, cells were collected in a 1.5 mL centrifuge tube, and 0.12 mL radio‐immunoprecipitation assay lysis buffer (Solarbio) was added to lyse the cells for 10 min. The lysates were centrifuged at 11,000×*g* for 20 min at 4 °C, and the supernatant (total protein) was transferred to a 0.5 mL tube. The total protein was quantified using a bicinchoninic acid protein quantification kit (Solarbio), and 10 µL protein samples (3.5 mg/mL) were prepared in the loading buffer. Samples (total protein) were separated using 10% sodium dodecyl sulfate polyacrylamide gel electrophoresis (PAGE). The separated proteins were transferred from the gel to a polyvinylidene difluoride (PVDF) membrane (Absin, China). The PVDF membrane was blocked with 5% skimmed milk for 3 h and then incubated with primary antibodies against β-catenin (1:1000), MGMT (1:1000), and β-actin (1:3000) (Cell Signaling Technology, Danvers, MA, USA) overnight at 4 °C. The PVDF membrane was washed three times with tris-buffered saline with 0.1% Tween 20 (TBST) and incubated with goat anti-rabbit IgG HRP-conjugated secondary antibody (Cell Signaling Technology) at 20 °C for 1 h. The PVDF membrane was washed with TBST, and 200 μL of enhanced chemiluminescent solution was added dropwise for color development. Imaging was performed under a gel imager. The ImageJ software (Version 1.48, Rawak Software Inc., Stuttgart, Germany) was used to analyze the density of the protein bands.

### Immunofluorescence analysis

Sterilized coverslips were placed in a six-well plate, and 5 × 10^5^ cells per well were added. Cells were cultured for an appropriate time and fixed with 4% paraformaldehyde (Beyotime Biotechnology, China). The wells were rinsed with PBS, and 1 mL 0.1% Triton X-100 (Sigma-Aldrich) was added to each well for 10 min. The wells were rinsed again with PBS, and goat serum (Tianhang, China) was added dropwise to the coverslips and incubated for 1 h. The serum was blotted with absorbent paper, and diluted β-catenin (1:100) or MGMT (1:50) antibodies were added dropwise onto the coverslip. Coverslips were incubated overnight at 4 °C. Slides were washed with PBS, and diluted fluorescent secondary antibody (Invitrogen, Carlsbad, CA, USA) was added dropwise to the coverslip. Coverslips were incubated for 1 h, washed with PBS, and added with Prolong Gold Antifade Reagent (Beyotime Biotechnology) and 4′,6-diamidino-2-phenylindole (Solarbio) dropwise. Slides were incubated for 5 min in the dark and mounted overnight at room temperature. The next day, the slides were imaged using an ECLIPSE Ts2R-FL fluorescence microscope (Nikon, Japan).

### Real-time fluorescence quantitative polymerase chain reaction (RT-FqPCR)

Cells were collected in a 1.5 mL tube and the total RNA was extracted with a Trizol kit (Sigma-Aldrich) following the manufacturer’s protocol. The miScript II RT Kit (Qiagen, Duesseldorf, Germany) was used to reverse transcribe total RNA into cDNA. cDNA was amplified using miR-214-5p primers and SYBR Premix Ex Taq II (Takara Bio Inc., Japan) on an ABI 7500 RT-FqPCR machine (Applied Biosystems, Foster City, CA, USA). The sequence of the miR-214-5p and U6 primers are as follows: miR-214-5p forward, 5′-ACACTCCAGCTGGGACAGCAGGCACAGAC-3′; miR-214-5p reverse, 5′-CTCAACTGGTGTCGTGGA-3′; U6 forward, 5′-GCTTCGGCAGCACATATACTAAAAT-3′; and U6 reverse, 5′-CGCTTCACGAATTTGCGTGTCAT-3′.

### Bioinformatics analysis

miRcode (http://www.mircode.org/index.php/) was used to predict the probability of miR-214-5p binding sites in *CTNNB1* and conservation of the binding sites among species. RNA22 v2 (https://cm.jefferson.edu/) was used to predict complementary binding sites in miR-214-5p and *CTNNB1*.

### Transfection

LN-18/TR cells were seeded in 10 cm dishes at 1 × 10^6^ cells/dish. The following day, DMEM containing miRNA mimic (50 nM; Thermo Fisher Scientific, Waltham, MA, USA) or miRNA inhibitor (120 nM; Thermo Fisher Scientific) and the corresponding negative control (NC) were prepared. Transfection was performed following the manufacturer's instructions. Cells were cultured for 24 h, and the medium was replaced. After 24 h, cells were harvested for RT-FqPCR to verify the level of miRNA.

### Dual luciferase reporter gene experiment

The 3′UTR of *CTNNB1* mRNA was linked downstream to the firefly luciferase reporter gene in the pmirGLO vector plasmid (Ke Lei Biotechnology Co., Ltd, China) to obtain a wild-type (wt) plasmid. In addition, the complementary paired sequences of the 3′UTR of *CTNNB1* mRNA and miR-214-5p seed region were mutated and linked downstream to the firefly luciferase reporter gene of the pmirGLO vector plasmid to obtain a mutant (mut) plasmid. LN-18 cells were seeded in a 24-well plate, and the experiment was divided into the control group (transfected with pmirGLO vector plasmid and pmirGLO vector plasmid + miR-214-5p mimic), wt group (transfected with wt plasmid and wt plasmid + miR-214-5p mimic), and mut group (transfected with mut plasmid and mut plasmid + miR-214-5p mimic). The plasmid transfection kit (Ke Lei Biotechnology Co., Ltd) and luciferase reporter test kit (Ke Lei Biotechnology Co., Ltd) were used for plasmid transfection and luciferase activity detection, respectively, according to the manufacturer's instructions.

### Flow cytometry

After drug treatment, cells were collected in a 10 mL tube and resuspended in 1 × Binding Buffer (Solarbio) at a concentration of 1 × 10^6^ cells/mL. The cells were mixed quickly and thoroughly. The cell suspension (100 µL) was transferred to a flow tube. To this, fluorescein isothiocyanate Annexin V and propidium iodide (Solarbio) were added according to the manufacturer's instructions and incubated for 20 min. Apoptosis was analyzed with an Accuri C6 Plus flow cytometer (BD Bioscience, San Jose, CA, USA).

### Animal experiments

The experiment was approved by the ethics committee of Hubei University of Chinese Medicine. Animal experiments were conducted in accordance with the Declaration of Helsinki and the ARRIVE guidelines. LN-18/TR cells were inoculated into the right armpit of BALB/c nude mice (HFK Bioscience Co., Ltd, China) at 5 × 10^6^ per mouse. When tumors grew to approximately 100–150 mm^3^, animals were divided into groups as follows, with six animals in each group: control, TMZ (20 mg/kg/d, ip), C3G (10 mg/kg/d, ip) + TMZ (20 mg/kg/d, ip), miR-214-5p agomir (1 mg/kg/3d, iv; Thermo Fisher Scientific) + TMZ group (20 mg/kg/d, ip). C3G or miR-214-5p agomir treatment was performed 4 h before TMZ administration. The experiment was carried out for 26 days, and the tumor long diameter, tumor short diameter, and nude mouse body weight were recorded three times per week. At the end of the experiment, all nude mice were euthanized, and the tumor was stripped and weighed. Tumor volume was calculated as follows: tumor volume (mm^3^) = long diameter × short diameter^2^/2.

### Statistical analyses

The GraphPad Prism software (Version 8.4.0, GraphPad Software Inc., San Diego, CA, USA) for Windows was used to analyze the data. All results are shown as means ± standard deviations. Comparisons between two groups were performed by a *t*-test; comparisons between multiple groups were performed by one-way analysis of variance followed by the least significant difference-*t* test for multiple comparisons. *P* < 0.05 was considered significant.

### Ethics declarations

We confirm the study was carried out in compliance with the ARRIVE guidelines. All methods were carried out in accordance with relevant guidelines and regulations.

## Supplementary Information

Supplementary Figure S1.

Supplementary Information.

## Data Availability

All data generated or analyzed during this study are included in this published article.
