# Supplementary figures and images for: Cyanidin-3-O-glucoside inhibits the β-catenin/MGMT pathway by upregulating miR-214-5p to reverse chemotherapy resistance in glioma cells
Source: Sci Rep. 2022 May 11;12:7773. doi: 10.1038/s41598-022-11757-w (PMC9095653; doi:10.1038/s41598-022-11757-w)

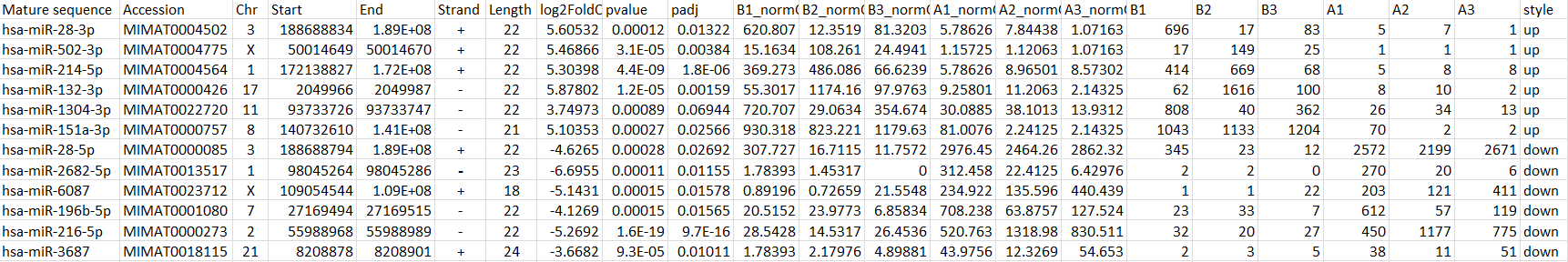

Supplement: Supplementary file 1 — Supplementary Figure S1. [file 41598_2022_11757_MOESM1_ESM.tif]

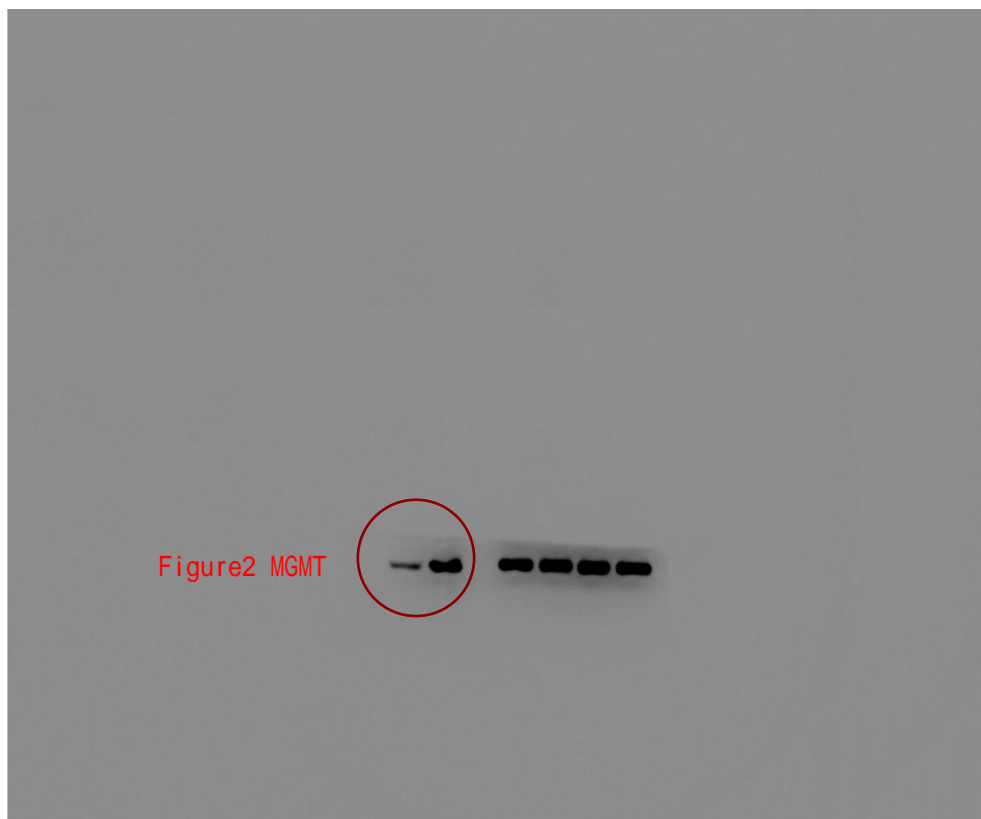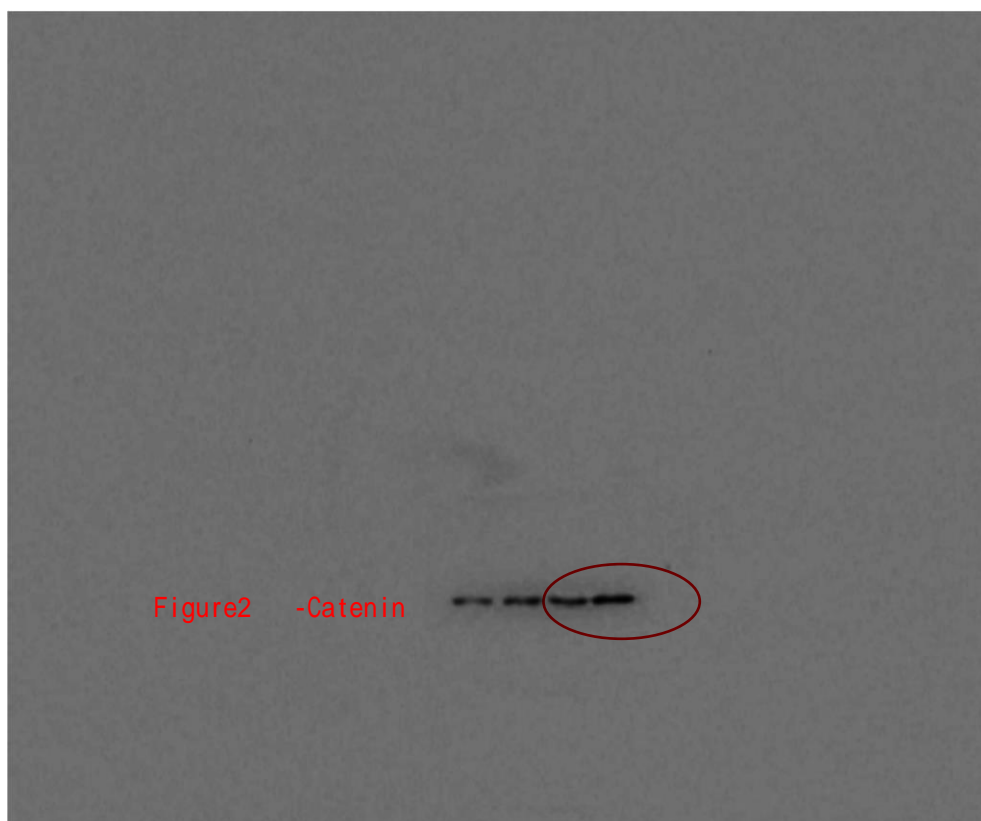

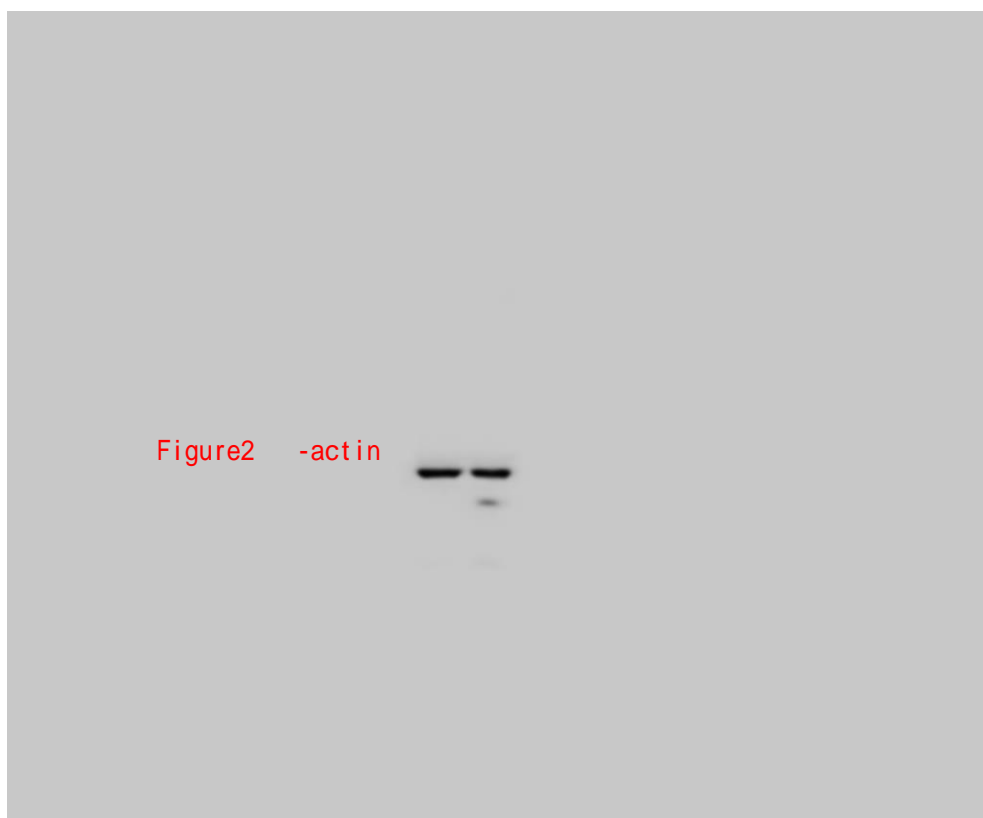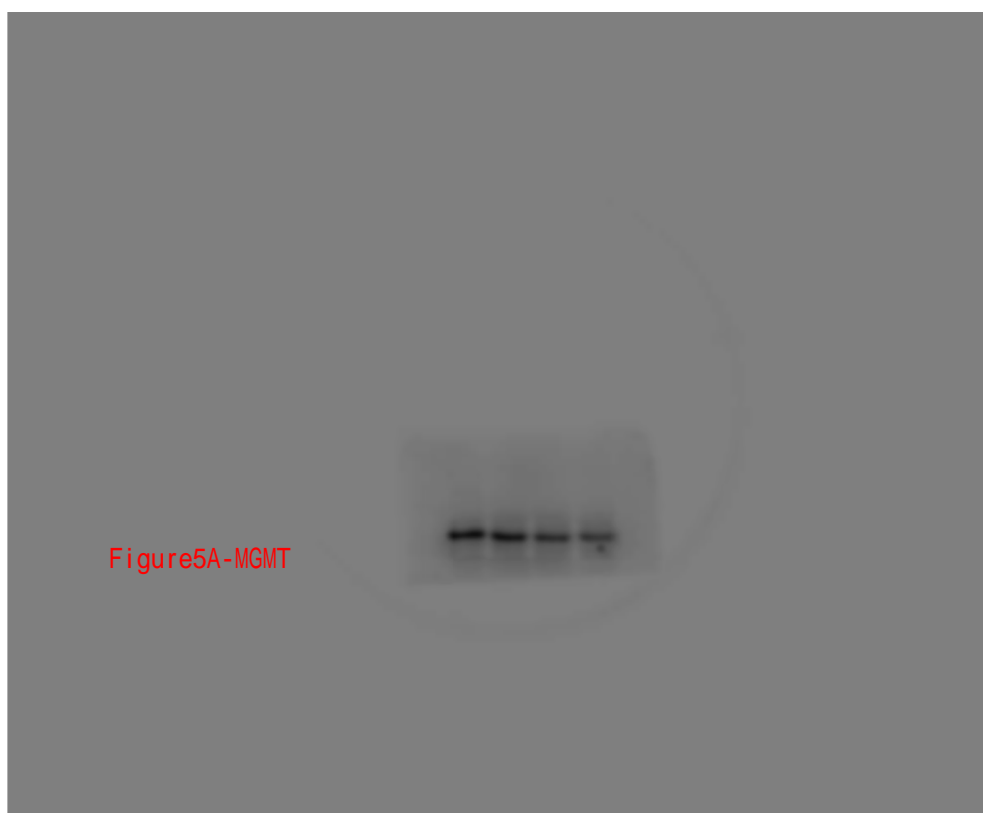

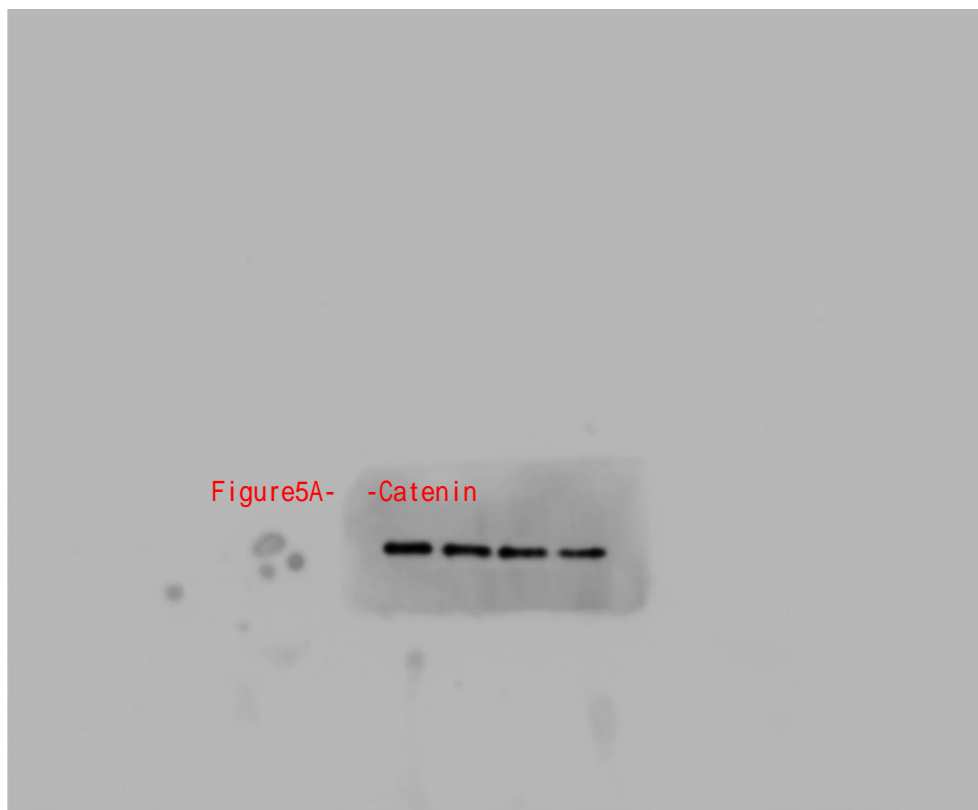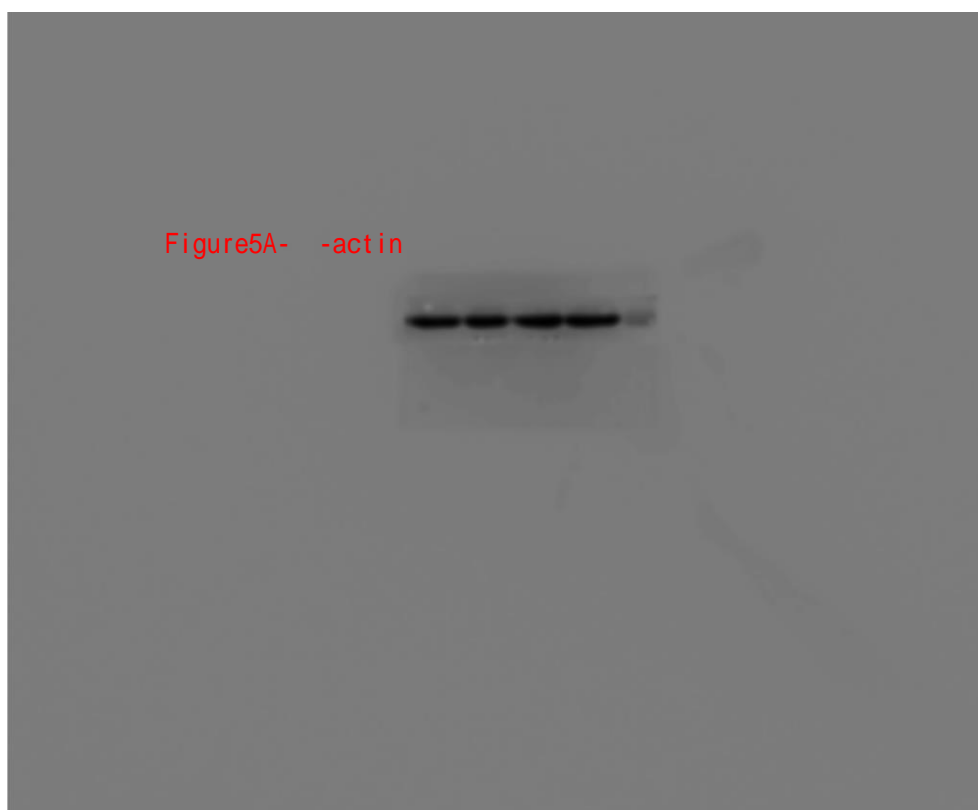

Figure5D- -Catenin

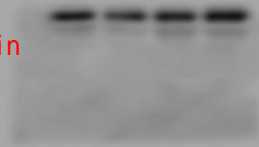

Figure5D-MGMT

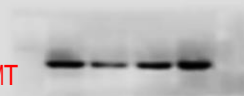

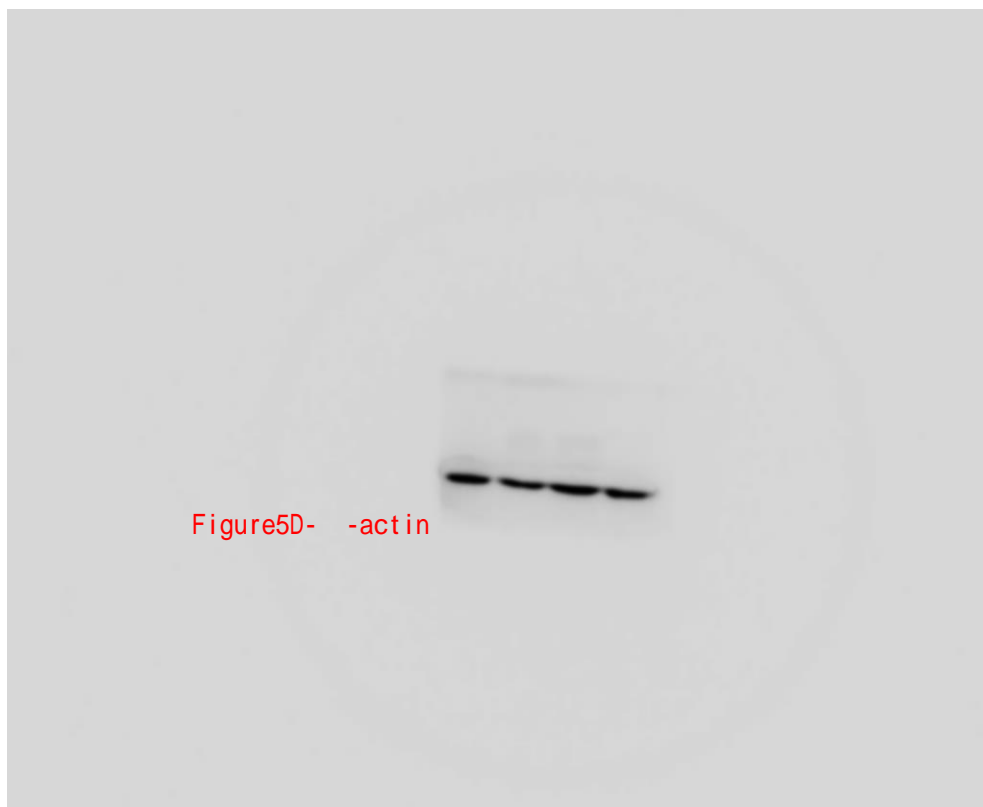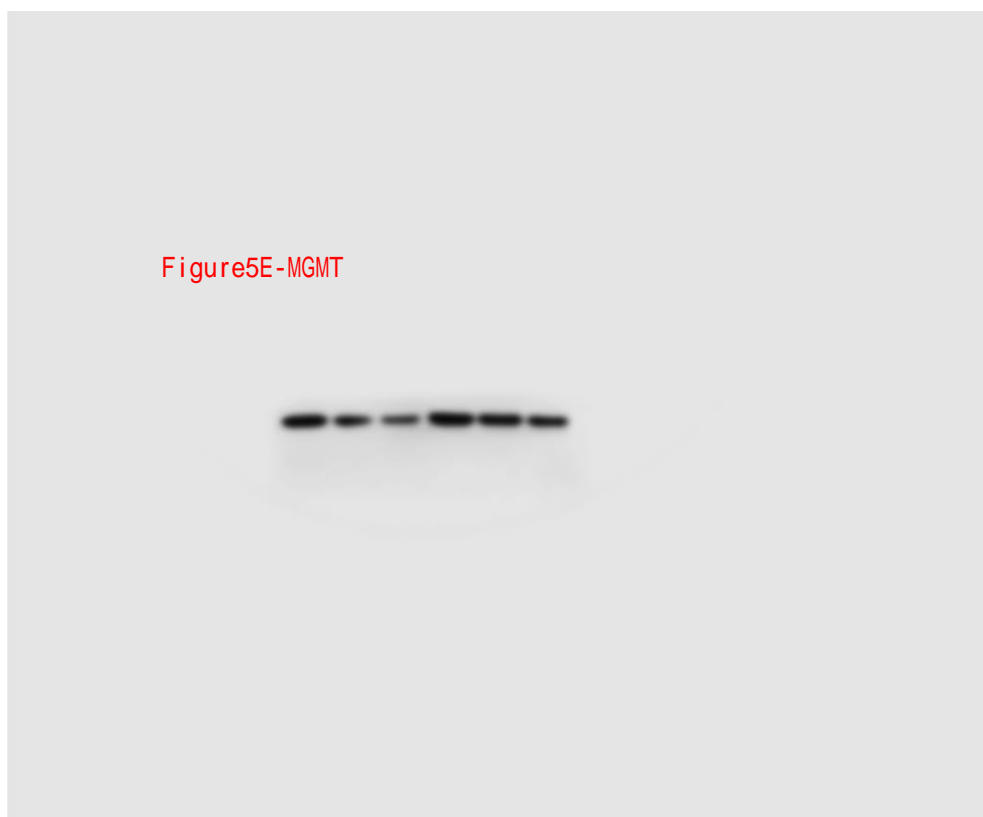

Figure5E-  $\beta$ -Catenin

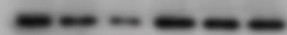

Figure5E-  $\beta$ -actin

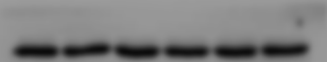

Supplement: Supplementary file 2 — Supplementary Information. [file 41598_2022_11757_MOESM2_ESM.pdf]
